# Outcome of prenatal depression and risk factors associated with persistence in the first postnatal year: Prospective study from Rawalpindi, Pakistan

**DOI:** 10.1016/j.jad.2006.10.004

**Published:** 2007-06

**Authors:** Atif Rahman, Francis Creed

**Affiliations:** a Department of Child and Adolescent Psychiatry, University of Manchester and Human Development Research Foundation, Islamabad, Pakistan; b Department of Psychological Medicine, University of Manchester, United Kingdom

**Keywords:** Postnatal depression, Mental health, Depression women, Developing countries, Pakistan

## Abstract

**Background:**

Rates of prenatal and postnatal depression in developing countries are high. Prolonged depression during the postnatal period is associated with impaired infant growth and development. Little is known about the factors predicting the persistence of prenatal depression beyond the first few postnatal months.

**Methods:**

From a sample of 701 women in a rural sub-district of Pakistan, the Schedule for Clinical Assessment in Neuropsychiatry (SCAN) was used to identify those with ICD-10 depressive disorder in the third trimester of pregnancy (*n* = 160). Depressed women were re-assessed at 3, 6 and 12 months postnatal. Persistently depressed women (depressed at all time points) were compared with the remainder. Psychiatric symptoms, disability and life events were measured using the Self-Reporting Questionnaire (SRQ), Brief Disability Questionnaire (BDQ), and a modified Life Events Checklist.

**Results:**

Of 129 mothers who completed follow-up, 73 (56%) were depressed at all points of assessment. These persistently depressed mothers had higher SRQ and BDQ scores prenatally and had experienced more life events in the year preceding the third pregnancy trimester than the mothers whose depressive disorder resolved (none received treatment). Persistent depression was significantly associated also with poverty, having 5 or more children, an uneducated husband and lack of a confidant or friend. On multivariate analysis, higher SRQ score and poverty during pregnancy predicted persistent depression.

**Limitations:**

The sample was from one rural sub-district only. We did not assess the women for physical conditions such as anaemia and thyroid-deficiency.

**Conclusion:**

Women who are poor and have more psychological symptoms during pregnancy are more likely to remain depressed one year after giving birth. This study highlights the need for developing mechanisms of early identification and suitable psychosocial interventions to minimise the damaging effects of persistent postnatal depression in poor communities.

## 1 Introduction

The rate of postnatal depression in developing countries ranges from 16% to 35% (Ghubash and Abou-Saleh, 1997; Patel et al., 2002; Aydin et al., 2005; Cooper et al., 1999) and is a major contributor to the ‘burden of disease’ in these countries. Depression around childbirth is associated with low birth weight and impaired weight gain in the first year of the infant's life (Rahman et al., 2004). The outcome is worse in infants whose mothers remained persistently depressed from the third trimester throughout the first postnatal year. It is, therefore, important to predict which mothers, depressed prenatally, are likely to remain depressed throughout the next year.

Relatively fewer studies have examined the long-term outcome of postnatal depression. A recent review of studies from developed countries concluded that in about 30% women with postnatal depression, symptoms persist for up to a year after giving birth (Goodman, 2004). There are no studies exploring the long-term outcome of postnatal depression in developing countries. Such longitudinal studies identify the course of depressive disorder and the factors that promote persistence thus helping to formulate effective preventive and treatment strategies (Weich and Araya, 2004). They can also help in targeting limited health resources in developing countries towards those at the greatest risk of poor outcome.

In an earlier paper (Rahman et al., 2003a,b), we assessed 632 women in rural Pakistan and found that 160 (25%) of the sample had ICD-10 Depressive disorder. In this paper, we present the results of the one-year follow-up of those mothers who were depressed in the third trimester of pregnancy.

## 2 Method

### 2.1 Study area, subjects and sampling

**Fig. 1:**
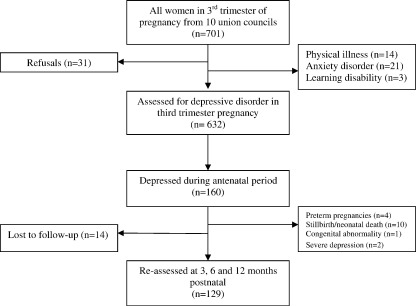
Sampling profile.

The study was carried out in a rural sub-district of Rawalpindi, Pakistan. This is a mainly agrarian low-income rural area about 60 km south-east from the city of Rawalpindi. All married women aged 17 to 40 in their third trimester of pregnancy (*n* = 701) were identified from 10 Union Councils (each consisting of 5–10 villages; total population 150,000) over a period of 4 months (Fig. 1). Subjects were identified by obtaining official lists from 120 government-employed Lady Health Workers (LHWs) working in the area, who routinely collect data on new pregnancies. Six-hundred and seventy out of 701 (95%) agreed to take part. Written informed consent was obtained from all subjects after the procedure had been fully explained. Fourteen (2%) were excluded because of a physical illness or complication of pregnancy, 21 (3%) had anxiety disorder and 3 (0.5%) had learning disability and were excluded. Out of the remaining, 160 were diagnosed with ICD-10 Depressive Episode, giving a prevalence rate of depressive disorder in the prenatal period (T1) of 25%. Out of these 160, four had infants born prematurely and were excluded from this study. Two mothers discontinued due to severity of depression. Ten mothers had stillbirths or neonatal deaths, and 1 newborn had a congenital abnormality and were excluded. Fourteen subjects dropped out of the study. Thus, one hundred and twenty-9 mothers were assessed at 3 months (T2), 6 months (T3) and 12 months (T4) postnatal.

### 2.2 Data collection

Mental state assessments were carried out at all time points by two trained and experienced clinicians using the Schedules for Clinical Assessment in Neuropsychiatry (SCAN), developed by the World Health Organization as an internationally validated semi-structured interview generating ICD-10 diagnoses of Depressive Disorder (World Health Organization, 1994a). All interviews were carried out after translation, back-translation and cultural adaptation of the interview schedule using an established procedure (Rahman et al., 2003a,b). Interrater reliability was established prior to the study when both interviewers independently assessed 20 women (10 had clinical depression) and agreed on the diagnosis of 19 (*κ* = 0.90).

Psychological symptoms were assessed at T1 by the same interviewers using the Self-Reporting Questionnaire (SRQ-20) (World Health Organization, 1994b). This consists of twenty items designed to identify psychological symptoms associated with anxiety and depression. Each item has a yes/no answer. The time span refers to the individual's feelings over the past 30 days. Each item is scored 0 or 1. The maximum score is therefore 20.

Disability in mothers was assessed at T1 using the Brief Disability Questionnaire (BDQ) (VonKorff et al., 1996). This is an 8-item questionnaire that rates current problems in carrying out daily activities on a scale of 0 (not at all) to 2 (definitely), with a maximum score of 16. This instrument has been validated in a 15-center cross-national, multilingual study (VonKorff et al., 1996).

Socio-demographic variables (age, education, employment, family structure and composition) were assessed at T1 by the same interviewers using a specially designed Personal Information Questionnaire (PIQ). Education was categorised into no education versus at least four years primary education. Four years of schooling was chosen as a cut-off because many Pakistani female children attend primary school for four years, after which many children from low-income families stop attending. Family structure was categorised into nuclear family (parents and children only) or extended family (three generations, or one or both parents with married sons, their wives and children).

Socioeconomic status was assessed at T1 by inquiring if the household was in debt and by asking Lady Health Workers, who lived in the same locality and had intimate knowledge of the families being studied, to rate the household on a 5-point Likert scale ranging from 1 (richest) to 5 (poorest). A single dichotomous variable of ‘poverty’ was created by combining these 2 measures, i.e., subjects who were both in debt and rated below 3 on the socioeconomic 5-point Likert scale were classified as being poor.

Maternal financial empowerment within the household was measured at T1 by asking the mothers if they were given a lump-sum amount of money for day-to-day household expenses, and whether they could take independent decisions about its use. Mothers who answered ‘yes’ to both questions were classified as financially empowered within the household. Social support was assessed by inquiring if the woman received any support during pregnancy from relatives or friends.

A brief list of life events and difficulties was administered at T1. These were derived from the Life Events and Difficulties Schedule (LEDS) (Brown and Harris, 1989), a semi-structured instrument that measures events and difficulties experienced during the previous year. LEDS has been translated and culturally adapted for use in the study area (Husain et al., 2000). Based on the data from this study, 9 types of events or difficulties that accounted for the majority of the severe events and difficulties reported in that population were used in a modified semi-structured interview. We recorded only those events and difficulties which rated as severe according to the Brown and Harris Rating Scale. In order to do this we discussed the context in which they occurred with the local lady health worker (who lived in the same community and had intimate knowledge of the families being studied).

### 2.3 Statistical analysis

All analyses were carried out with STATA, version 7 (StataCorp., 2001) Subjects who were depressed at all 4 time points (persistent cases) were compared with the rest (non-persistent cases). *T*-test was used to compare psychological symptoms, disability and life event scores. Univariate analyses (relative risk, Fisher's two sided exact *p*) was performed between potential risk factors and chronic depression. Associations were considered significant at the 5% level. The simultaneous effects of the measured risk and protective factors on persistent depression were analyzed using logistic regression analysis, including all the variables studied in the model (listed in Table 1).

The study was approved by the ethics committees of Rawalpindi Medical College, Pakistan and University of Manchester, UK.

## 3 Results

### 3.1 Sample characteristics

129 depressed women completed the one-year follow-up. Their average age was 27.5 years (SD = 5.3). All were married, the average age of marriage being 20 years (SD = 2.8). Forty-four percent were uneducated; only 4% were employed outside the home. Eighty-eight percent of the fathers were employed and about 22% of them were absent from home for 6 months or more due to employment in the cities. The average reported monthly family income was 2500 rupees (US$42). Sixty-seven percent of the families were rated below 3 on the 5-point socioeconomic scale by the LHWs and 53% of the households were in debt. Sixty-two (48%) were in debt *and* rated below 3 by the LHWs and were classified as ‘poor’ in this study.

Nine percent were primigravid, 16% already had one child, 21% two children, 17% three children and the remaining 37% had three or more. Thirty-seven percent lived in nuclear families (parents and children only) while the remaining lived in extended families (three generations, or one or both parents with married sons, their wives and children). Fifty-seven percent delivered at home with a traditional birth attendant, 98% without any reported complication. The gender of newborns was equally distributed.

Of the 129 women, 121 (94%) were depressed at 3 months, 98 (76%) at 6 months and 80 (62%) at 12 months. Eighty out of 129 (62%) mothers depressed during the third trimester of pregnancy were still depressed at 12 months postnatally but 7 of these had not been depressed at 6 months; thus 73 (57%) were depressed at all time points.

A comparison of the 73 women who had persistent depressive disorder with the remainder on scores of the Self-Reporting Questionnaire, Brief Disability Questionnaire and Life Events Checklist is shown in Table 1. Persistent depression was significantly associated with SRQ score and BDQ score, and weakly associated with Life Events score.

Unadjusted relative risks with other factors are shown in Tables 2 and 3. Persistent depressive disorder was associated with having an uneducated husband, family size (5 or more children), poverty and lack of a confiding relationship.

Independent predictors of persistent depression selected by multiple logistic regression were: high score on the SRQ in the third trimester of pregnancy (odds ratio (OR) 1.3, 95% CI 1.1 to 1.6, *p* < 0.01) and poverty (OR 3.1, 95% CI 1.2–8.4, *p* > 0.05) (Table 4).

## 4 Discussion

To our knowledge, this is the first study from the developing world exploring the course of prenatal depression and factors predicting its persistence during the first year of the newborn's life. The study was community based and used standardised and valid instruments to diagnose depression. Depression around childbirth is a serious public health problem in south Asia, affecting about one in four women (Patel et al., 2002; Rahman et al., 2003a,b). Recent studies also provide strong evidence that maternal depression is associated with poor growth in infants living in poor communities in developing countries (Rahman et al., 2004; Patel et al., 2004), and the outcome is worse in infants of mothers with persistent depression. In developed countries, maternal depression is associated with long-term cognitive, emotional and behavioural problems in children, and the impact is worse where the depressive episode is severe or prolonged (Grace et al., 2003; O'Connor et al., 2002). Therefore, prolonged maternal depression has serious consequences not only for the mother but also for infant growth and development.

The main findings of this study are that over half of mothers depressed in the third trimester of pregnancy continued to be depressed one year after giving birth. In similar studies from developed countries, depressive levels have been found to decrease steadily over time. In an early prospective study from USA, O'Hara et al. (1984) followed up 99 women from second trimester of pregnancy to 6 months after giving birth, and found that although almost one half of the subjects had depressive scores that would place them in the mildly depressed range during the second trimester, less than 12% of the subjects were in the mildly depressed range at the 9-week and 6-month follow-ups. In a more recent American study, Campbell and Cohn (1997) followed up 70 women meeting the criteria for clinical depression at 2 months postnatal and found that at 4 months postnatal, 48% continued to be depressed; at 6 months 30% and at 12 months, 24% continued to meet the criteria for depression.

Beeghly et al. (2002) followed up 106 women with high depression scores at 2 months postnatal and found that 35% and 31% continued to have high scores at 6 and 12 months respectively. Rubertsson et al. (2005) followed up a national cohort of Swedish women and found that out of 333 women with high depression scores in early pregnancy, 79 (24%) continued to have high scores at one year postpartum. A follow-up study of an Australian cohort from early pregnancy till five years after birth suggested that in the majority of women who experienced depressed mood after birth, the symptoms were not severe and did not continue beyond a few weeks (Najman et al., 2000). In our study, prevalence of antenatal depression is high and comparatively fewer women recover in the first year after childbirth.

The low rate of recovery could be due to the adverse circumstances experienced by many women in developing countries. Persistent depression was associated with several factors that preceded the birth: poverty, already having 5 or more children, an uneducated husband, and lack of a friend or confidant. Similar factors have been reported in studies from poor communities in developed countries. Horowitz and Goodman (2004) found that women still depressed two years after giving birth were more likely to be poor and have less social support. Bernazzani et al. (1997) found that lower occupational status, lower income, prenatal depression and stressful life events in 12 months prepregnancy were associated with depression persisting at 6 months postnatal. Yonkers et al. (2001) found that persistent postnatal depressive symptoms were linked with the presence of other young children at home. In the current study, multivariate analyses suggested poverty to be the major predictor of persistence of depression after severity of depressive symptoms in the prenatal period was adjusted for.

The gender of the newborn did not predict persistence. In south Asia, giving birth to a female infant was found to be associated with postnatal depression (Patel et al., 2002), especially in mothers who already had more than two girl-children (Rahman et al., 2003a,b). The preference for male children is deeply rooted in South Asia. Women are often blamed for the birth of girls. However, this study suggests that the effects of having a female child on the mother's mood may only be transient. Similarly, lack of social support during pregnancy (in terms of assistance provided in daily activities) was not associated with persistence while lack of a confidant or friend was. It may be that there are qualitative differences in the type of social support that predicts a worse outcome of depression, and studies using better measures of social support may be required.

While only physically healthy mothers were included in the study, we did not include any physical measures to screen mothers for common physical problems such as anaemia or iodine-deficiency. However, chronic malnutrition in the mothers indicated by a low body-mass index was not significantly associated with persistent depression. Other limitations include a relatively small sample size and the fact that all the women came from one sub-district of Rawalpindi.

Cross-sectional epidemiological studies in Pakistan suggest that the prevalence rate of depressive disorder in women is high even in the non-postnatal period (Husain et al., 2000; Mirza and Jenkins, 2004). Other longitudinal studies would be required to try and understand the origins of depression in these women, which may well pre-date the first pregnancy, or even have its onset in adolescence. However, the postnatal period assumes importance because of the impact of maternal depression on the infant's development. Thus, early identification of mothers whose depression is likely to persist and providing extra support could improve outcomes in both mothers and their infants.

The strongest predictor of persistent depression in this study was a high score on the SRQ-20 in the third trimester pregnancy. Recent meta-analyses suggest that psychological symptoms (mainly depression and anxiety) during pregnancy, along with past history of psychiatric illness are primary risk factors for postnatal depression (O'Hara and Swain, 1996; Beck, 2001). The few studies that have explored the long-term outcome of postnatal depression suggest that depressive symptoms during pregnancy (Bernazzani et al., 1997; Verkerk et al., 2003) not only predict early postnatal depression but are also associated with persistence of depression. The utility of the SRQ-20 in predicting persistent depression suggests that this questionnaire, which has been specifically developed for use in primary care in developing countries, could also serve as a useful screening instrument during the antenatal period.

## Figures and Tables

**Table 1:** Comparison of prenatal measures of psychological symptoms, disability and life events

Measure	Mean non-persistent	Mean persistent	Mean difference (95% CI)	*t*-test *p*
SRQ score	9.4	13.0	3.6	< 0.01
			2.3–4.8	
BDQ score	5.8	7.5	1.7	< 0.01
			0.7–2.6	
Life events score	1.4	1.9	0.5	< 0.05
			0.08–0.8	

**Table 2:** Unadjusted relative risks of persistent depression with risk and protective factors (*N* = 129)

Risk or protective factor	Non-persistent *N* = 56	Persistent *N* = 73	Unadjusted relative risk	95% CI	*p*value*
≥ 30 years of age	14 (25)	30 (41)	1.3	1.0–1.8	0.06
≤ 20 years of age	6 (11)	7 (10)	0.9	0.6–1.6	1.0
Not educated	22 (39)	35 (48)	1.2	0.9–1.6	0.3
Financially empowered	26 (46)	26 (36)	0.8	0.6–1.2	0.2
Primigravida	8 (14)	4 (5)	0.6	0.3–1.3	0.1
BMI < 18.5	14 (25)	13 (18)	0.8	0.5–1.6	0.4
Husband not educated	5 (9)	23 (32)	1.7	1.3–2.2	< 0.01
Husband unemployed	4 (7)	12 (16)	1.4	0.9–1.9	0.1
Husband away for > 6 months	13 (23)	15 (21)	0.9	0.7–1.4	0.8
Nuclear family	17 (30)	31 (42)	1.2	0.9–1.7	0.1
Having 2 or more children under 7	35 (62)	52 (71)	1.2	0.8–1.7	0.3
Having 5 or more children	8 (14)	21 (29)	1.4	1.1–1.8	0.05
Having 2 or more girl-children	24 (43)	40 (55)	1.2	0.9–1.7	0.2
Female gender of newborn	25 (45)	40 (55)	1.2	0.9–1.6	0.2
Lack of social support	38 (68)	48 (66)	0.9	0.7–1.3	0.8
Poverty	20 (36)	42 (58)	1.4	1.1–1.9	< 0.05

*Fisher two sided exact *p*.

**Table 3:** Unadjusted relative risks of persistent depression with life events in the previous year (*N* = 129)

Life event	Non-persistent *N* = 56	Persistent *N* = 73	Non-adjusted relative risk	95% CI	*p* value*
Significant other made reduntant	10 (18)	10 (14)	0.8	0.5–1.4	0.6
Housing difficulties	1 (1)	1 (1)	0.8	0.2–3.5	1.0
Major arguments, relationship difficulty	6 (11)	13 (18)	1.3	0.9–1.8	0.3
Serious marital problems	3 (5)	4 (5)	1.0	0.5–1.9	1.0
Bereavement	15 (27)	18 (25)	0.9	0.7–1.4	0.8
Major illness in family	20 (36)	31 (42)	1.1	0.8–1.4	0.4
Social role change	19 (34)	35 (48)	1.2	0.9–1.60	0.1
Problems with the law	1 (2)	3 (4)	1.3	0.7–2.4	0.6
Loss of friend or confidant	6 (11)	19 (26)	1.5	1.1–1.9	< 0.05

*Fisher two sided exact *p*.

**Table 4:** Estimates of simultaneous effect of risk factors* on persistent depression through multiple logistic regression (*n* = 129)

Risk/protective factor	Odds ratio	Standard error	95% confidence interval	*p* value
SRQ score	1.3	0.1	1.1–1.6	< 0.01
BDQ score	1.0	0.1	0.8–1.3	0.9
Life events score	1.1	0.2	0.7–1.6	0.8
Husband uneducated	2.6	1.6	0.7–9.1	0.1
Having 5 or more children	2.1	1.4	0.5–7.6	0.3
Poverty	3.1	1.6	1.2–8.4	< 0.05

*Only those variables are shown that had significant association on univariate analysis. All other variables not shown remained insignificant.
